# Hyperchylomicronemia causes endothelial cell inflammation and increases atherosclerosis

**DOI:** 10.21203/rs.3.rs-5451391/v1

**Published:** 2024-11-25

**Authors:** Maria Concepcion Izquierdo, Ainara G. Cabodevilla, Debapriya Basu, Dimitris Nasias, Jenny E. Kanter, Winnie Ho, Jana Gjini, Edward A. Fisher, Jeffrey Kim, Warren Lee, Karin E. Bornfeldt, Ira J. Goldberg

**Affiliations:** 1 Division of Endocrinology, Diabetes and Metabolism, New York University Grossman School of Medicine, New York, NY 10016; 2 Division of Metabolism, Endocrinology and Nutrition, Department of Medicine, University of Washington, Seattle, WA 98109; 3 Keenan Centre for Biomedical Research, St. Michael’s Hospital and Division of Critical Care, Department of Medicine, University of Toronto, Canada; 4 Division of Cardiology, Department of Medicine, New York University Grossman School of Medicine, New York, NY; 5 Comparative Medicine Research Unit, University of Louisville School of Medicine, Louisville, KY

**Keywords:** Triglycerides, lipoproteins, lipoprotein lipase, lesion, BCA, aortic root

## Abstract

The effect of increased triglycerides (TGs) as an independent factor in atherosclerosis development has been contentious, in part, because severe hypertriglyceridemia associates with low levels of low-density lipoprotein cholesterol (LDL-C). To test whether hyperchylomicronemia, in the absence of markedly reduced LDL-C levels, contributes to atherosclerosis, we created mice with induced whole-body lipoprotein lipase (LpL) deficiency combined with LDL receptor (LDLR) deficiency. On an atherogenic Western-type diet (WD), male and female mice with induced global LpL deficiency (i*Lpl*^−/−^) and LDLR knockdown (*Ldlr*^*kd*^) developed hypertriglyceridemia and elevated cholesterol levels; all the increased cholesterol was in chylomicrons or large VLDL. After 12 weeks on a WD, atherosclerotic lesions both in the brachiocephalic artery and the aortic root were more severe in i*Lpl*^−/−^*/Ldlr*^*kd*^ mice compared to the control *Ldlr*^*kd*^ mice. One likely mechanism for this is that exposure of the aorta to hyperchylomicronemia led to endothelial cell inflammation. Thus, our data show that intact chylomicrons contribute to atherosclerosis, explain the association of postprandial lipemia and vascular disease, and prove that hyperchylomicronemia is not benign.

## INTRODUCTION

Elevated low-density lipoprotein cholesterol (LDL-C) is a major causal factor for cardiovascular disease (CVD). Whether elevated levels of triglyceride (TG)-rich lipoproteins (TRLs) cause CVD, as conjectured more than 50 years ago^1,2^, is debated. TRLs include intestinally-derived chylomicrons, hepatically-secreted very low-density lipoprotein (VLDL), and their remnants created by partial loss of TG via actions of capillary-associated lipoprotein lipase (LpL). Although remnant lipoproteins are thought to be atherogenic, nascent TRLs are postulated to not be atherogenic due to a failure to penetrate the arterial wall^3^. This often-quoted observation was made in diabetic rabbits that have extraordinarily large circulating particles with a lipid composition, unlike human and mouse chylomicrons^3^.

In the past decade our understanding of how LDL crosses the arterial endothelial lining has evolved. Two endothelial cell (EC) receptors—scavenger receptor-BI (SR-BI) and activin-like kinase 1 (ALK1)—are required for LDL transcytosis^4,5^; EC deficiency of these receptors reduces atherosclerosis^5,6^. SR-BI also serves as a receptor for HDL^7^ and chylomicrons^8,9^.

LpL is the rate-limiting enzyme hydrolyzing TG in circulating chylomicrons and VLDL. GWAS analysis strongly associates heterozygous mutations in LpL and other genes regulating lipolysis with CVD risk^10^. While many clinicians have suggested that chylomicrons are not atherogenic despite their levels correlating with CVD risk^11^, this assumption is based on limited data suggesting that LpL-deficient patients do not develop atherosclerosis. This conclusion may be incorrect, as atherosclerosis is documented to occur in these patients despite their very low levels of circulating LDL-C^12,13^. In hyperchylomicronemic mice, small lipid-filled arterial lesions have been reported in older mice with genetic deficiency of LpL or its binding protein, glycosylphosphatidylinositol anchored high-density lipoprotein binding protein 1 (GPIHBP1)^14^. However, as with human LpL deficiency, these mice have very low LDL-C levels^15^. Whether hyperchylomicronemia in settings in which LDL is not reduced would increase vascular disease is unknown.

To overcome the marked reduction in LDL-C in LpL-deficient mice, we created mice with induced deletions in both LpL and the LDL receptor (LDLR). Even with limited lipolysis, these mice had a marked increase in LDL-C on a Western diet (WD). Our data show that defective lipolysis and the creation of severe hypertriglyceridemia cause greater lesion development. These studies resolve the long-standing controversy on the atherogenicity of large TRLs and debunk the hypothesis that chylomicrons are benign.

## RESULTS

### Combined LpL and LDLR deficiency does not dramatically reduce LDL-C in mice.

Whole-body LpL knockout mice die within the first 1–2 days after birth^16^. To create LpL-deficiency in adult mice, we generated inducible LpL knockout mice (i*Lpl*^−/−^) by crossing LpL floxed mice (*Lpl*^fl/fl^) with β-actin-driven tamoxifen-inducible-Cre (Mer/Cre/Mer) transgenic mice^15^. *Lpl*^fl/fl^ and i*Lpl*^−/−^ mice were given intraperitoneal injections of 4-hydroxytamoxifen in corn oil at a dose of 40 mg/kg body weight/day for five consecutive days. i*Lpl*^−/−^ mice analyzed three weeks after the last tamoxifen injection show an approximately 95% reduction in LpL gene expression and an 80–90% decrease in LpL activity in heart, skeletal muscle, and white adipose tissue, and a >90% reduction in plasma LpL activity^15^. This level of LpL activity loss in mice is similar to that found in LpL-deficient humans with familial hyperchylomicronemia syndrome when activity is measured using similar methods^16,17^. Loss of LpL in mice fed a regular chow diet resulted in a large increase in plasma TG to approximately 400–500 mg/dL; plasma cholesterol was not increased (**Supplement Figure 1A and B**). These levels of circulating TG are similar to those found with very low-fat diets in many human LpL-deficient patients^18,19^. Lipid analysis of plasma lipoprotein fractions following density gradient ultracentrifugation showed increased TG and cholesterol in d<1.006 g/mL lipoproteins (chylomicrons and VLDL, designated as TRLs) in i*Lpl*^−/−^ mice (**Supplement Figure 1C** and **D**). LDL-C and high-density lipoprotein (HDL)-C were lower in i*Lpl*^−/−^ mice (**Supplement Figure 1E-F**). LDL TG was increased (**Supplement Figure 1G**), while HDL TG was less than 5 mg/dL and at the lower limits of the assay (not shown). These data are consistent with a role for LpL in the formation of LDL and HDL during TRL lipolysis^20^.

**Figure 1.**
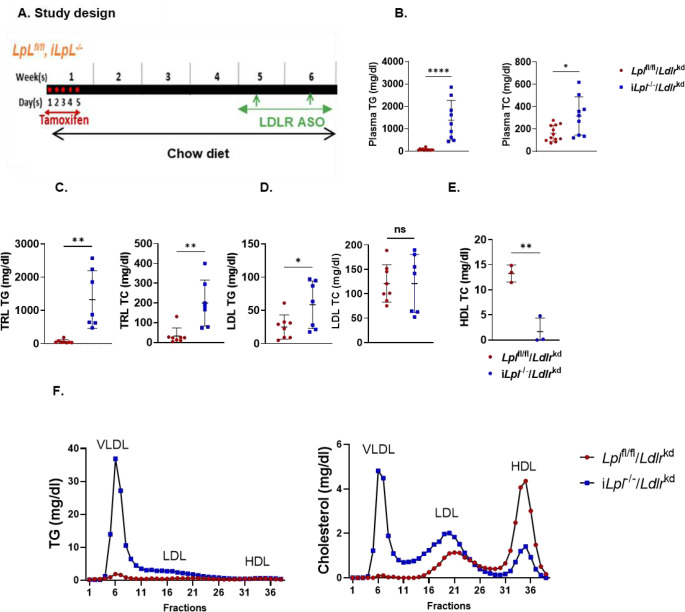
Lipoprotein profiles in *Lpl*^*−/−*^/*Ldlr*^*kd*^ mice. **A.** *Lpl*^*flox/flox*^/beta actin Cre mice (6 weeks) were injected intraperitoneally with tamoxifen (of 40 mg/kg × 4 days). The mice were maintained on a chow diet and 4 weeks later received LDLR ASO. Two weeks later blood was obtained to assessed lipoproteins and the mice were then switched to a WD. **B.** Plasma triglyceride (TG) and total cholesterol (TC) levels on chow. **C.** Lipoproteins were isolated by ultracentrifugation. VLDL (d<1.006 g/mL) TG and cholesterol. **D.** LDL (d 1.019–1.053 g/mL) TG and cholesterol. **E.** HDL (d 1.063–1.22 g/mL) cholesterol **F.** Sera from 3–5 mice were pooled, 200 μL was analyzed by FPLC and TG and cholesterol measured.

We next assessed the plasma lipid phenotype of i*Lpl*^−/−^ mice in the absence of the hepatic LDLR. We induced LpL deletion with tamoxifen treatment as described above and three weeks later knocked down the hepatic LDLR using an antisense oligonucleotide (ASO) at a dose of 5 mg/kg body weight/week for another two weeks (Figure 1A); these combined deficient mice are denoted i*Lpl*^−/−^/*Ldlr*^kd^. Our previous publication describes this ASO-mediated hepatic LDLR KD in detail^21^. As expected, LpL deficiency markedly increased plasma TG and cholesterol in *Ldlr*^kd^ mice (Figure 1B). This was also found with isolation of TRL-TG and TRL-C as shown in Figure 1C. Unexpectedly, as compared to mice with normal LDLR expression (shown in **Supplement Figure 1E**), knockdown of the LpL did not lead to a marked reduction in LDL-C in i*Lpl*^−/−^/*Ldlr*^kd^ mice. In fact, the LDL-C levels were similar levels to those found in i*Lpl*^fl/fl^/*Ldlr*^kd^ mice that had normal TRL lipolysis (Figure 1D). LDL-TG was greater in i*Lpl*^−/−^/*Ldlr*^kd^ mice. In a subgroup of mice, we also assessed HDL-C, which was reduced as would be expected due to reduced HDL production with the lack of lipolysis (Figure 1E). Consistently, FPLC analysis showed a marked increase in VLDL-TG and VLDL-C, a moderate shift to larger LDL size, and a marked reduction in HDL-C with LpL deficiency in the setting of LDLR KD (Figure 1F).

We next placed another group of mice on a Western diet (WD) (study design shown in **Supplement Figure 2A**). Not surprisingly, the total TG levels were markedly increased, in some mice to over 10,000 mg/dL (**Supplement Figure 2B**); total cholesterol was also increased. As expected the increases were primarily due to more TG and cholesterol in TRLs (**Supplement Figure 2C**); LDL-C levels were increased as compared with chow-fed mice (**Supplement Figure 2D,** left panel). As before, HDL-C was lower in i*Lpl*^−/−^/*Ldlr*^kd^ mice (**Supplement Figure 2E**, left panel). Both LDL and HDL TG content were greater (**Supplement Figure 2D and 2E,** right panel). These changes in isolated lipoproteins were similar to those found by FPLC (**Supplement Figure 2F**) and, surprisingly, show that mice create LDL even with a marked reduction in TRL lipolysis.

### LpL knockdown has only minor effects on LDL-C levels in *Ldlr*^kd^ mice fed WD for 12 weeks.

**Figure 2.**
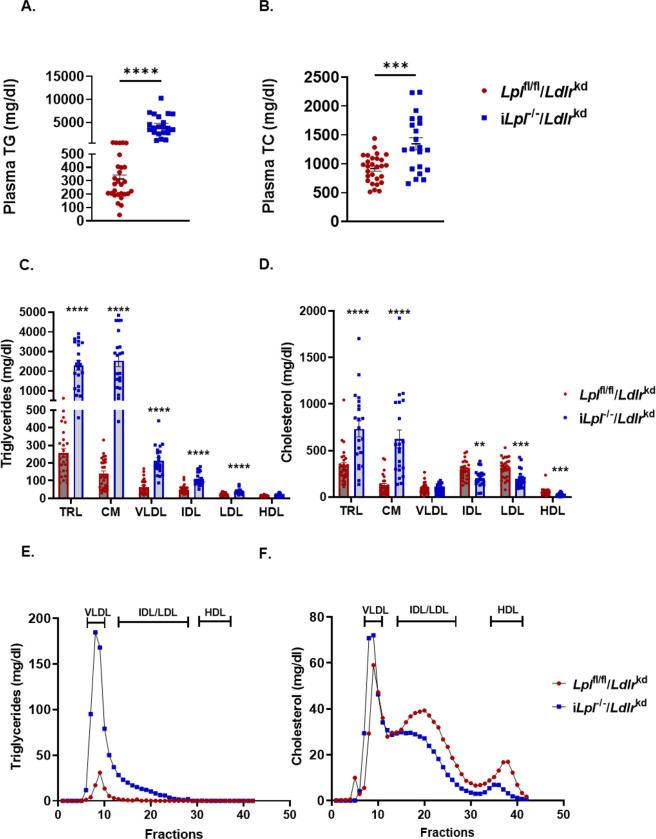
Plasma triglyceride-rich lipoprotein cholesterol and triglyceride levels are increased in *iLpl*^*−/−*^ mice. *Lpl*^fl/fl^ and i*Lpl*^−/−^ after 12 weeks of LDLR knockdown using PCSK9 AAV and WD feeding. **A.** TG levels. **B.** Total TC levels. **C,D.** Distribution of TG and cholesterol levels in plasma fractionated by size-exclusion chromatography. **E.** Plasma TGs distribution on ultracentrifuge-fractionated lipoproteins. **F.** Plasma cholesterol distribution on ultracentrifuge-fractionated lipoproteins. n= 21–28/group. Data are presented as the mean ± SEM. * p<0.01; ** p<0.005, *** p<0.001, **** p<0.0001 vs *Lpl*^fl/fl^ mice by Student’s test.

To study the effects of increased TRLs on atherosclerosis in mice that were LDLR deficient, we fed male mice a WD and employed a less arduous method for longer-term reduction of the LDLR, AAV expression of proprotein convertase subtilisin/kexin type 9 (PCSK9). With this protocol, both TG and cholesterol increased (Figure 2 A and B); the rise in TG was much greater with LpL deficiency, to >1000 mg/dL. We further fractionated the TRLs using a short time spin of 30 min to isolate chylomicrons (Figure 2 C and D). The majority of the TRLs were isolated by this short spin, i.e. most of the cholesterol increase in the LpL-deficient mice was due to very buoyant TRLs denoted CM in the figure. As was seen with our ASO protocol, loss of LpL activity allowed continued production of LDL-C; LDL-C and intermediate-density lipoprotein (IDL)-C levels were only slightly lower than in controls. As expected, HDL-C levels were lower with LpL deficiency. These changes in lipoproteins were also seen by FPLC, which showed a marked increase in VLDL-TG, a similar small reduction in LDL-C, and a reduction in HDL-C (Figures 2E and F).

### Mice lacking LpL and LDLR have larger aortic root lesions.

**Figure 3.**
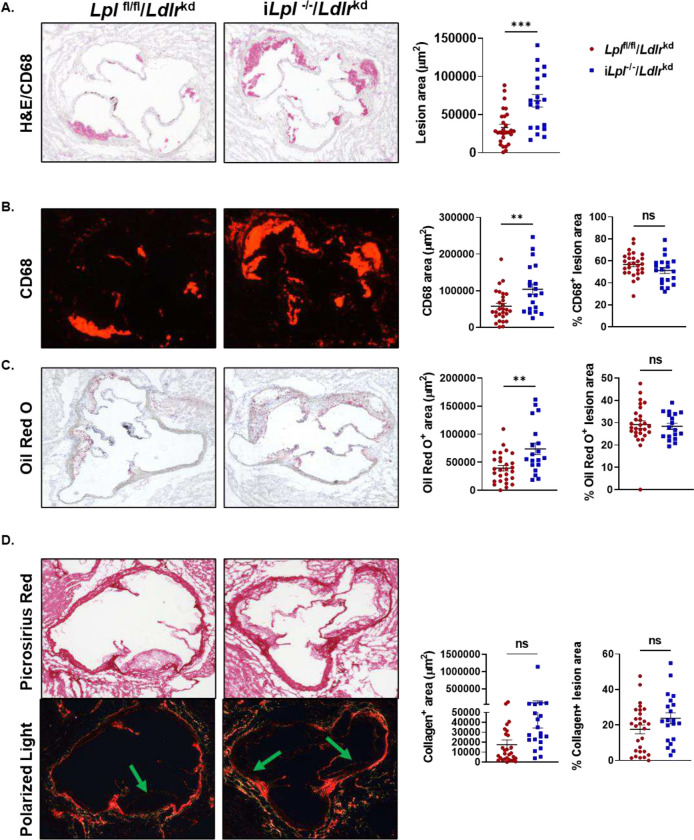
Aortic root lesions are larger in *iLpl*^*−/−*^*/Ldlr*^*kd,*^ mice. *Lpl*^fl/fl^ and i*Lpl*^−/−^ after 12 weeks of LDLR knockdown using PCSK9 AAV and WD feeding. **A. Left:** Representative histological analysis of cross sections of the aortic root stained with hematoxylin and CD68 from *Lpl*^fl/fl^ and i*Lpl*^−/−^ mice. **Right:** Quantification of plaque size. **B. Left:** Representative immunofluorescence staining of CD68 (macrophage marker) of cross sections of the aortic root stained from *Lpl*^fl/fl^ and i*Lpl*^−/−^ mice. **Right:** Quantification of macrophage content. Results expressed as mean ± SEM. **C. Left**: Representative staining of Oil Red O (ORO) of cross-section of the aortic root stained from *Lpl*^fl/fl^ and i*Lpl*^−/−^ mice. **Right:** Quantification of ORO. **D. Left**: Representative staining of Picrosirius red (bright field (top) and polarized light (bottom) of cross section section of the aortic root stained from *Lpl*^fl/fl^ and i*Lpl*^−/−^ mice. **Right:** Quantification of collagen content in atherosclerosis lesions. n=21–28 mice/group. Values are mean ± SEM.

The lipoprotein profile found with i*Lpl*^−/−^/*Ldlr*^kd^ allowed us to then ask whether increased TRLs affected atherosclerosis lesions, as the model did not suffer from a dramatic loss of LDL-C. Aortic root lesion analysis of mice consuming a WD for 12 weeks showed that i*Lpl*^−/−^/*Ldlr*^kd^ mice had significantly larger plaques than control *Ldlr*^kd^ mice (Figure 3A). Markers of plaque instability, lipid and collagen content as well as CD68 expression are shown in Figure 3B–D. Mice lacking LpL and LDLR exhibited greater absolute area occupied by macrophages (CD68+, Figure 3B) and increased lipid content (oil red O–ORO, Figure 3C), which were both accounted for by the larger lesions. Collagen content was not significantly different between mice lacking LpL and LDLr, as compared with the control group (Figure 3D). Therefore, as a percentage of the lesion area, macrophage, lipid and collagen in atherosclerotic lesions was similar between the two groups, indicating that while the lesions were larger, they did not appear to be less stable.

We next performed an analysis to determine whether circulating lipid levels correlated with lesion size. Neither chylomicron-, LDL-, IDL-, or HDL-C cholesterol levels correlated with lesion area (**Supplement Figure 3A-D**).

### LpL deficiency increased lesion size also in female mice.

We performed similar atherosclerosis studies using female mice. As with males, i*Lpl*^−/−^/*Ldlr*^kd^ mice had higher TG and cholesterol levels on WDs, but the TG increases were less pronounced than in males (**Supplement Figure 4A**). Like in males, the lipoprotein increases were restricted to chylomicrons and VLDL with decreases in IDL-, LDL-, and HDL-C (**Supplement Figure 4B**). Despite a slight (non-significant) reduction in LDL-C, hypertriglyceridemic female mice also had increased aortic root lesion size (**Supplement Figure 5A**).

### Male mice lacking LpL and LDLR have larger and more complex brachiocephalic artery (BCA) lesions.

**Figure 4.**
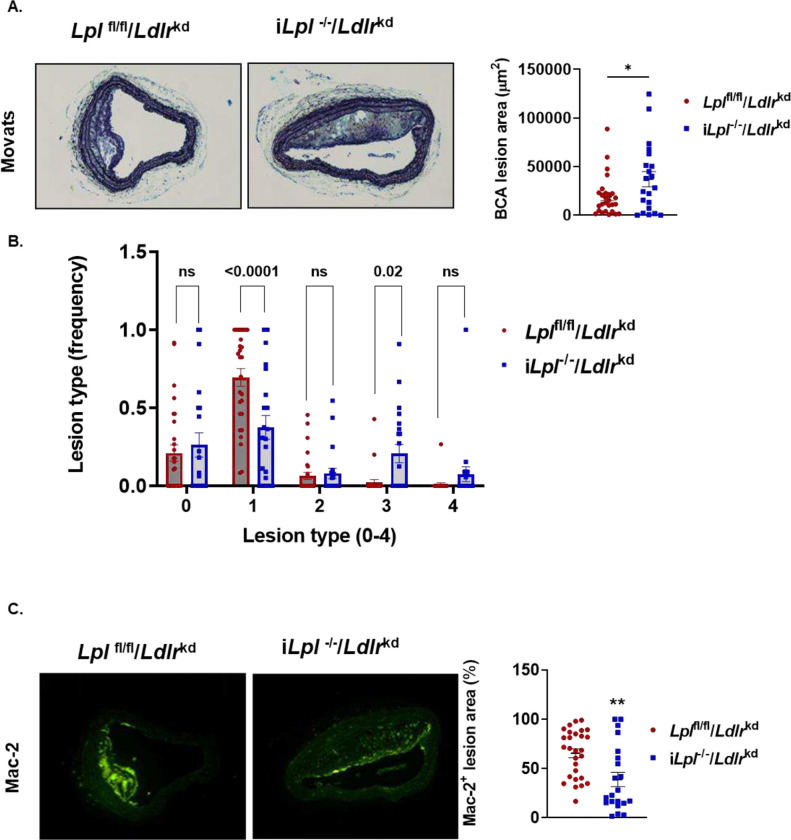
Lesions in the brachiocephalic artery (BCA) are bigger in *Lpl*^*−/−*^*/Ldlr*^*kd*^ mice. *Lpl*^fl/fl^ and i*Lpl*^−/−^ after 12 weeks of LDLR knockdown using PCSK9 AAV and WD feeding. **A. Left**: Movat’s pentachrome stain of a representative BCA cross-section from *Lpl*^fl/fl^ and i*Lpl*^−/−^ mice. **Right:** Quantification of plaque size. **B.** Lesion scoring 0. No lesion; 1. Macrophages only lesions; 2. Macrophages + Extracellular matrix/GAG; 3. Macrophages + ECM + Collagen; 4. Macrophages + GAG + Collagen + Necrotic cores. n=21–28 mice/group. **C. Left:** Representative immunofluorescence staining of macrophage (Mac-2 positive) in BCA cross-section from *Lpl*^fl/fl^ and i*Lpl*^−/−^ mice. **Right:** Quantification of macrophage content. Data are presented as the mean ± SEM. * p<0.01; ** p<0.005 vs *Lpl*^fl/fl^ mice by Student’s test.

Atherosclerotic lesions were also evaluated in the BCA. Lesions at the maximal lesion site in male i*Lpl*^−/−^/*Ldlr*^kd^ mice were larger than those of the floxed control group (Figure 4A). BCA lesions were also more advanced in the i*Lpl*^−/−^/*Ldlr*^kd^ mice compared to the control group (Figure 4B). In particular, mice lacking LpL and LDLR had significantly fewer fatty streak lesions consisting of macrophages only (lesion type 1) and significantly more lesions consisting of macrophages with collagen and extracellular matrix accumulation (lesion type 3) than LDLR-deficient controls. Consistently, deficiency of LpL and LDLR led to a relative reduction in lesional macrophages, detected using Mac-2 staining (Figure 4C). These data suggest that systemic LpL deficiency overrides the known anti-atherogenic actions of macrophage-specific LpL deficiency^22,23^. Unlike the males, female i*Lpl*^−/−^/*Ldlr*^kd^ mice did not have greater atherosclerosis than non-hypertriglyceridemic flox/flox controls in the BCA (**Supplement Figure 5B**), perhaps due to their somewhat lower lipid levels.

### WD-fed LpL-deficient mice have no differences in circulating or arterial leukocytes.

Since the number of lesional macrophages was reduced in the BCA lesions and others have reported that macrophage LpL deficiency reduces circulating monocyte levels^24^, we evaluated whether differences existed in circulating WBCs. Flow cytometry analysis revealed no differences in circulating T cells, B cells, monocytes, or neutrophils between *Ldlr*^kd^ and i*Lpl*^−/−^/*Ldlr*^kd^ mice **(Supplement Figure 6**). We next collagenase digested the aortas and assessed the numbers and gene expression of arterial WBCs. The FACS analysis showed no differences in the percentage of monocytes, neutrophils, macrophages, eosinophils, or T or B cells between *Ldlr*^kd^ and i*Lpl*^−/−^/*Ldlr*^kd^ mice (**Supplement Figure 7**). These findings suggest that the larger lesions in LpL- and LDLR-deficient mice are not explained by increased numbers of circulating monocytes.

### Aortic gene expression at single-cell resolution. Aortic gene expression at single-cell resolution.

**Figure 5.**
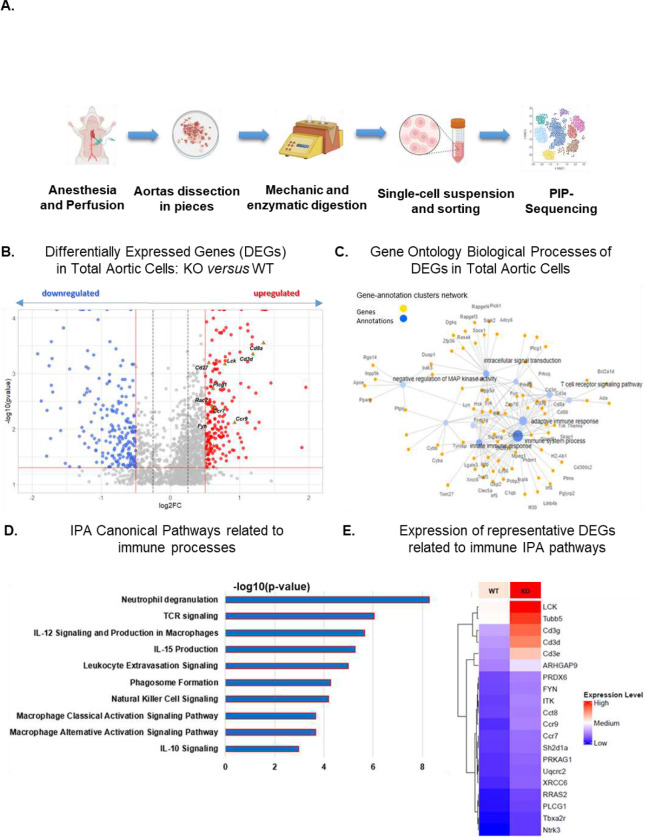
Single cell sequencing analysis of aortic cells. **A.** Schematic diagram of the experimental design to analyze mouse aortas with scRNA-seq technology. **B.** Volcano plot of differentially expressed genes (DEGs) in aortas. The x axis represents the log2-transformed fold change, and the y axis represents the p-value transformed in the negative log 10. Red dots represent the statistically significant (pvalue<0.05) upregulated genes in the LpL-deficient mice and the blue ones the downregulated ones (abdolute fold change >0.5). Labeled genes are highlighted with green asterisks inside triangle markers. **C.** Network of genes related to Gene Ontology Biological Processes enriched in aortic cells. Yellow nodes represent genes enriched in a category, while blue nodes correspond to Biological Processes. Node sizes are proportional to the −log10 of the adjusted p-value, reflecting the statistical significance of each enrichment. **D.** The bar chart displays the top enriched Canonical IPA pathways associated with immune response. Pathways shown were filtered based on their p-values (Fisher Exact Test) and include those comprising at least three genes involved in immunity. Each bar represents a pathway, with the bar height reflecting the level of significance for that pathway’s enrichment, providing a visual comparison of immune-related pathway involvement across the analyzed data. **E.** Heatmap of key Immune Response Genes across Groups. This heatmap displays representative genes involved in immune responses, as identified through IPA pathway analysis. Expression values are z-score transformed, ranging from −1 to 1, to highlight relative expression differences. Groups are defined as follows: WT (Wild Type) and KO (Knockout) for LpL deficiency.

Our data suggest that chylomicrons increase atherosclerosis. Having shown that these lipoproteins are internalized and degraded by ECs (8), we next tested whether LpL deficiency and its resultant hyperchylomicronemia lead to aortic inflammation. To assess change prior to atherosclerosis development, *Ldlr*^*−/−*^ mice with or without concomitant induced LpL deficiency were fed a WD for 2 weeks prior to harvesting the lower aortic arch. A schematic of this experiment is shown in Figure 5A. To explore how hypertriglyceridemia associates with aortic cell gene changes, we performed differential expression analysis of total aortic cells, shown in the volcano plot (Figure 5B and **Supplement Table 1**). We identified 371 differentially expressed genes when comparing the i*Lpl*^−/−^/*Ldlr*^kd^ group to the control, with the majority (259 genes) being upregulated and 112 downregulated in the LpL- and LDLR-deficient mice. Some of the top upregulated genes, known for their roles in immunity, are highlighted by distinct shapes (triangle) and colors (red-green) in the figure based on their fold change (>0.5) and pvalue (<0.05). Looking for biological interpretation in an unbiased fashion, a network analysis of Gene Ontology biological processes confirmed that most of the induced genes affected white cell response including innate immune response and components of the adaptive such as T cell receptor signaling pathway (Figure 5C). To further validate the bioinformatical evidence of enhanced inflammation, our Ingenuity pathway analysis (IPA) (Figure 5D and **Supplement Table 2**) indicated that innate immunity, neutrophil degranulation, macrophage activation, and other possible pro-atherogenic pathways were statistically significantly enriched. Some of the induced genes are related to T cytotoxic cells (Figure 5C) whose activity has been well documented in arherosclerosis progression ^25,26^. Also, we identified the downregulation of the gene *Traf3* whose protein inhibits NFκB and the downstream inflammatory cascade^27,28^. Among the genes categorized in IPA immune pathways were the lymphocyte-specific protein tyrosine kinase (LCK), genes of the CD3 complex in T lymphocytes (eg. CD3g) and the the C-C chemokine receptors *Ccr7* and *Ccr9* (Figure 5E and **Supplement Table 2**).

Single-cell sequencing of the cells from the aortas identified three cell clusters shown as a Uniform Manifold Approximation and Projection (UMAP) plot in **Supplement Figure 8A.** To identify each cluster, we applied a standard Seurat pipeline “FindMarkers” by using several canonical markers, plotting of genes across the clusters and literature mining to confirm biological releavance based on experimentally observed data. The aortic cluster (AC) 0 expressed markers of vascular smooth muscle cells (SMCs) such as *Vim, Tagln2, Myh9,* and *Acta2*^29^ (**Supplement Figure 8B and D**). AC-0 was characterized by the expression of several well-known proinflammatory markers such as *Il-1b*, *S100a8*, *S100a9,* and *Ccr2* implying aortic residual inflammation^30^. AC-1 comprises cells expressing immune-related markers such as *Rac2, NKg7, Ccr7, Cd8a,* and *Cd8b1* that are highly expressed in leukocyte subsets (immune cells)^30^ with cytotoxic function such as T CD8+ cells. AC-2 contained markers that are specific to aortic ECs such as *Cdh5, Vwf, Tek*, and the panmarker of ECs *Pecam1*^29,31^ (**Supplement Figure 8B and D**). Comparison of aortic single-cell RNA-sequencing data sets from WD–fed mice suggest that there is an expansion of AC-1 representative of immune cells in the double knockout mice **(Supplement Figure 8C).** Differential expression analysis in each cluster reveals an induction of immune responses as shown in the volcano plots and pathway analyses for cell type (**Supplement Figure 9 and Supplement Table 1**). Notably, we identified that the gene *Traf3* is not expressed in any cluster of double knockout mice but only in the controls (Figure 5B). *Traf3* codes an inhibitor of NFκB signaling that hinders a pro-inflammatory response^27^. These data indicate that the atherogenic effects of hyperchylomicronemia were not limited to a single cell type.

### Lipid particles from diabetic rabbits have an impaired ability to undergo endothelial transcytosis.

**Figure 6.**
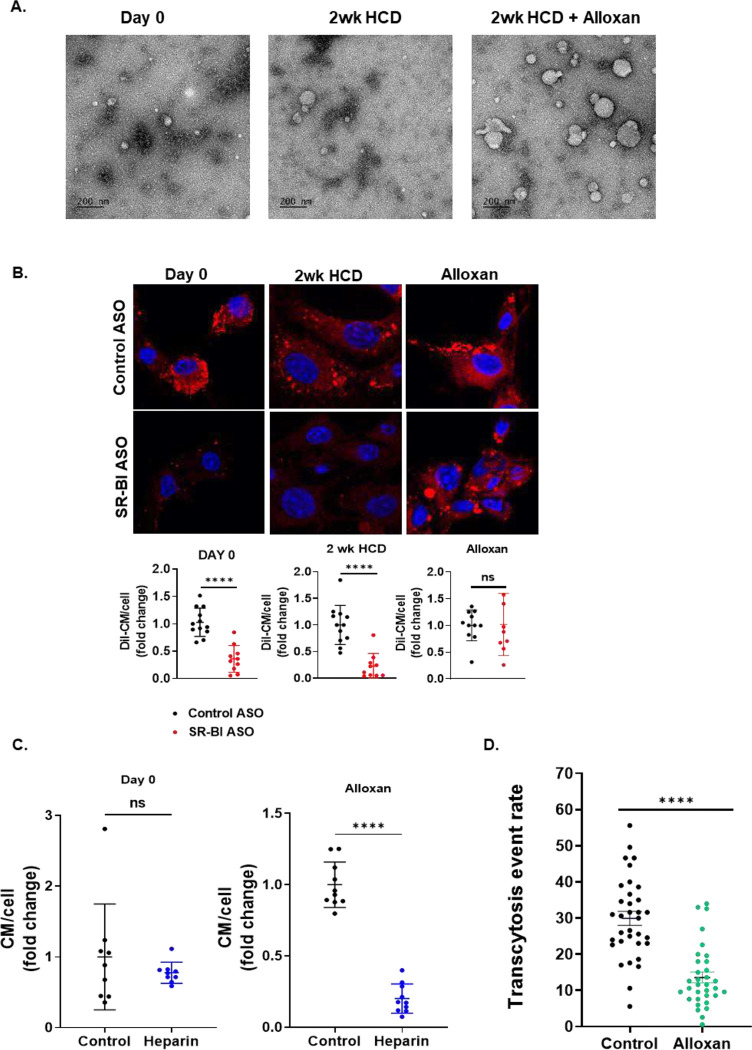
Lipid-rich particles from diabetic rabbits are defective in their interaction with ECs. Chylomicrons were isolated from the plasma of control, high-cholesterol diet fed (termed 2wk HCD), or alloxan-treated rabbits fed a high-cholesterol diet (termed Alloxan). **A.** Electron microscope images of chylomicrons obtained from control (left panel), high-fat diet-fed (middle panel), or alloxan-treated rabbits (right panel). **B.** Mouse aortic ECs were treated with control or SR-BI ASO for 48hs, deprived of serum overnight, then exposed to DiI-labeled rabbit chylomicrons for 30 minutes. Cells were then fixed and imaged using a Leica SP8 confocal microscope to detect intracellular chylomicrons. SR-BI depletion significantly reduced the uptake of chylomicrons obtained from control and high-cholesterol diet-fed rabbits (left and middle panels and graphs), but not of chylomicrons obtained from alloxan-treated diabetic rabbits (right panels and graph). **C.** Treatment with 10mg/ml heparin significantly reduced EC uptake of chylomicrons obtained from Alloxan-treated but not control rabbits. **D.** Chylomicrons from Alloxan-treated rabbits undergo significantly less transcytosis than those obtained from control rabbits, as monitored by TIRF microscopy. Data are presented as mean ± SD. **** p<0.0001 as compared to control, by Student’s t-test.

Our data show that chylomicrons have deleterious effects on ECs, which may drive atherosclerosis. This result contrasts with a widely accepted view that chylomicrons are not atherogenic because they fail to cross the EC barrier. Because studies supporting this hypothesis were performed in diabetic cholesterol-fed rabbits^3^, we tested whether the large lipid particles created in diabetic rabbits interacted with ECs in a similar manner to mouse and human TRLs. We isolated TRLs from control, high cholesterol-diet-fed, and diabetic cholesterol-fed rabbits. The giant particles created with diabetes are shown in Figure 6A. Although TRLs from control and non-diabetic cholesterol-fed rabbits were internalized by ECs via a process inhibited by SR-BI ASO, uptake of the enormous diabetic rabbit lipid particles was not affected by knockdown of SR-BI (Figure 6B). Moreover, unlike control chylomicrons, the uptake of these particles was reduced by low-dose heparin (Figure 6C), suggesting that they are associated with cell surface proteoglycans rather than SR-BI. We also assessed whether lipids from control and diabetic rabbit chylomicrons undergo transcytosis using total internal reflection fluorescence (TIRF) microscopy (Figure 6D). In agreement with the studies of Nordestgaard^3^, lipids from these giant particles do not cross the EC, unlike control chylomicrons.

## DISCUSSION

The role of LDL-C as a major causal factor for CVD is not in dispute, whereas the role of TRLs in atherogenesis is still debated. The multivariate origin and subclasses of TRL further add to the complexity of the question. To study this, we created mice with a combined deficiency of LpL and the LDLR to test whether nascent TRLs are atherogenic. On a chow diet, induced LpL deficiency led to reduced circulating LDL-C, but when combined with *Ldlr*^kd^ the mice had continued production of LDL. Despite a small reduction in LDL-C, i*Lpl*^−/−^/*Ldlr*^kd^ mice with hyperchylomicronemia had increased atherosclerosis. We then went on to provide mechanistic information on how chylomicrons cause more disease. Exposure of ECs to chylomicrons led to greater inflammation and increased expression of monocyte adhesion molecules. Thus, we show that vascular uptake of TRLs, as occurs during postprandial lipemia^8,32^, produces vascular toxicity. We were able to perform these studies because, despite LpL deficiency, which should have reduced VLDL to LDL conversion, i*Lpl*^−/−^/*Ldlr*^kd^ mice did not have almost complete LDL deficiency. Others have suggested that LDLR deficiency allows for direct hepatic production of LDL, i.e., hepatic secretion of LDL not derived from a VLDL precursor^33,34^. In vitro studies have shown that the LDLR can capture newly created LDL either before^33^ or after^34^ its release from hepatocytes. Thus, with LDLR KD a pool of LDL may have been secreted from the liver, explaining the production of LDL in i*Lpl*^−/−^/*Ldlr*^kd^ mice. Another option is that some residual but low levels of LpL were sufficient to allow continued VLDL to LDL conversion and that hepatic LDL clearance due to LDLR-deficiency contributed to the high LDL levels in LpL- and LDLR-deficient mice. Mice with a deficiency of both LpL and the LDLR had hypertriglyceridemia and increased cholesterol levels; the cholesterol increase was due to chylomicrons, as assessed by ultracentrifugation. Despite the slight reduction in circulating LDL-C and the lack of LpL expression in macrophages (see below), atherosclerosis was increased. These data are consistent with the human studies showing that multiple genes that reduce TRL clearance and lead to greater postprandial hypertriglyceridemia increase coronary artery disease^35^.

Although advanced atherosclerosis may be associated with defects in the arterial endothelial barrier, recent work strongly supports the hypothesis that arterial LDL accumulation is mediated by EC luminal wall receptors (reviewed in^36^). One receptor implicated in LDL transcytosis across ECs is SR-BI^4^. When SR-BI was selectively knocked out in ECs, atherosclerosis was reduced^5^. SR-BI is also a receptor for chylomicrons^8^. For this reason, we tested whether increased atherosclerosis in hypertriglyceridemic mice could be due to changes in EC biology. Exposure of cultured ECs and aorta to chylomicrons led to an inflammatory profile including upregulation of genes known to attract circulating monocytes, ICAM, and VCAM.

Our observations showing increased atherosclerosis in mice with hyperchylomicronemia contrast with the assumption that large TRLs are not atherogenic because they are unable to penetrate the arterial wall^3^. This hypothesis evolved from studies of diabetic high cholesterol diet-fed rabbits that have enormous lipoproteins with an unusual ratio of TG/cholesterol^37^. Using radioactive tracer methods, the investigators of these studies reported that, unlike LDL, these huge rabbit lipoproteins were unable to contribute lipids to arterial wall plaques. The reason for this was postulated to be that the particles were too large to passively cross the EC barrier. We agree with this conclusion, but for a different reason. Our understanding of the mechanisms allowing lipoprotein entry into the arterial wall has been altered in the past decade. It is unlikely that any lipoproteins passively cross the arterial EC barrier^36^. Rather, TRLs and LDL must interact with EC receptors to initiate transcytosis. Unlike normal chylomicrons from humans, mice, and rabbits, diabetic cholesterol-fed rabbit particles do not interact with SR-BI. For this reason, they can not deliver lipids to the sub-endothelial space. Therefore, the reduction in atherosclerosis in diabetic rabbits is not solely due to the size of the TRLs, but to their failure to bind to EC lipoprotein receptors.

LpL deficiency has been created in mice and reproduces many aspects of human familial chylomicronemia syndrome (FCS). This includes the development of severe hypertriglyceridemia. Like humans with FCS, generalized LpL knockout mice are born without any gross abnormalities but unlike humans these mice die shortly after birth^16^, likely due to hypoglycemia^38^. An LpL-expressing adenovirus has been used to rescue the LpL knockout mice through the suckling phase. On a chow diet for 20–24 weeks, these mice develop small foam cell-containing lesions in the aortic arch^39^. Mice lacking GPIHBP1, the capillary EC LpL-binding protein, also have extreme hyperchylomicronemia and when older develop lesions in their aortic arches and BCAs^14^. These two severely hypertriglyceridemic mice models have a relatively modest amount of arterial lipid accumulation compared to hypercholesterolemic mice^14,37^, likely due to the low LDL-C levels. As implied by these studies, chylomicrons, not just remnants, promote atherogenesis.

Our lab and others have shown that macrophage-specific deficiency of LpL in mice reduces atherosclerotic lesions^23,40^, highlighting the atherogenic nature of macrophage LpL in mice. Thus, macrophage LpL deficiency in the i*Lpl*^−/−^/*Ldlr*^kd^ model should have reduced atherosclerosis, making the increase in lesion size even more impressive.

In human LpL deficiency, assessment of atherosclerosis and the importance of TRLs is complicated by the very low plasma LDL-C levels. Consistent with this, there are reports documenting no atherosclerosis in arteries of a >60-year-old women and a 53-year-old man with LpL deficiency^41,42^. On the other hand, another report described four patients with LpL deficiency and premature atherosclerosis^12^. A more recent report described two siblings with LpL deficiency and CVD^13^ and a survey of patients from a large medical center found that 50% of patients with FCS developed vascular disease by age 60^43^ . The reasons for these divergent vascular effects are unknown but could depend on diet, the type and degree of LpL mutation, or other confounding factors known to modulate atherogenesis.

Genetic and function studies of the lipolysis pathway suggest that defective clearance of postprandial lipoproteins increases risk for CVD. Heterozygous LpL mutations track with greater CVD^44^. Moreover, increased postprandial lipemia^45^ and damaging mutations in the LpL associate with an increased presence of coronary artery disease^46^. Conversely, LpL mutations that increase LpL activity, which should be associated with less postprandial lipemia, are protective ^47^. Other genes that regulate TG metabolism such as the LpL inhibitors angiopoietin-like protein 3 and 4 (ANGPTL3, ANGPTL4)^48,49^, apolipoprotein C-3 (APOC3) and apolipoprotein A-5 (APOA5) are also linked to the risk of CVD^50,51^. Our data provide an explanation for these gene associations.

Despite the many indications that increased TRLs drive atherosclerosis, clinical studies selectively reducing these particles and showing a reduction of CVD events have failed to materialize. This has led some to conclude that TRLs are not atherogenic, in part, because studies using fibric acid drugs to reduce TG levels did not reduce CVD events^52,53^. However, a more recent study treating moderately hypertriglyceridemic patients with pemafibrate, which reduced TRLs but increased LDL, showed no benefit, i.e., cholesterol within TRLs was equally atherogenic to LDL^54^. This conclusion is similar to that in our study showing that TRLs, even large chylomicron-like particles, are atherogenic. Our results are also consistent with the large Danish studies showing that TRLs in non-fasting samples correlate with CVD risk^55^.

In conclusion, our studies provide experimental and mechanistic evidence consistent with the human genetic and lipoprotein association studies showing that hypertriglyceridemia leads to more CVD^55^. In addition, they demonstrate that chylomicrons are not benign. Although circulating apolipoprotein B lipoproteins provide cholesterol and other lipids that deposit within the arterial wall, lipoproteins also affect vascular health by affecting EC biology. Consistent with recent genetic, population, and clinical intervention studies, our data should dismiss the notion that large TRLs and postprandial lipemia are benign.

## METHODS

### Mice and diets:

All procedures were approved by the Institutional Animal Care and Use Committees at New York University Langone Health (animal protocol #160907 “Lipoprotein Lipase and ApoB”). 12–18-week-old, male and female mice were maintained in a temperature controlled (25°C) facility with a 12-h light/dark cycle. Male mice were used for a majority of experiments to avoid introducing variability due to hormonal changes in female mice. Mice were given free access to water and food, except when fasting blood specimens were obtained. Mice were either fed a rodent chow diet or an atherogenic WD (Dyets Inc. catalog no. 101977, 0.3% cholesterol), as indicated.

### LpL deficiency:

Global inducible LpL-knockout mice were generated by crossing floxed Lpl (Lplfl/fl) mice (47) with β-actin driven tamoxifen-inducible Cre (MerCreMer) transgenic mice (Jackson Laboratory) to obtain the β-actin-MerCreMer/LpLfl/fl offspring designated as i*Lpl*^−/−^ mice, as previously described (48). Mice received intraperitoneal (i.p.) injections of tamoxifen (Toronto Research Chemicals, #T006000) or 4-hydroxytamoxifen (Tocris) in corn oil (Sigma) at a dose of 40 mg/kg BW/day for 4 times, administered every other day.

#### LDLR knockdown:

We used two methods to reduce LDLR in hypertriglyceridemic mice. Mice with a knockdown of LDLR are denoted *Ldlr*^kd^. For studies, the LDLR was knocked down using antisense oligonucleotides (ASOs) provided by IONIS Pharma, as reported previously (19). Because atherosclerosis studies require a longer LDLR knockdown, PCSK9 AAV was purchased from University of Pennsylvania Vector Core. 1X10^12^ genome copies was injected per mouse via retro-orbital injection in a volume of 100 μl adjusted with sterile PBS.

### Blood sampling:

All blood samples were collected after 4 hours of fasting unless specified. Blood was collected from the retro-orbital sinus of mice using heparinized micro-capillary tubes. Blood was centrifuged at 10,000 g for 10 minutes for cell removal and collection of the plasma, which was then used for lipid measurements and/or frozen at −80°C.

### Lipid measurements:

Total cholesterol (TC) was measured using Infinity Total Cholesterol Reagent (#TR13521, Thermo Scientific, Waltham, MA). Plasma TG was measured using Infinity Triglycerides (#TR2242, Thermo Scientific, Waltham, MA).

#### Lipoprotein fractionation:

Lipoproteins were analyzed by both ultracentrifugation and fast-protein liquid chromatography (FPLC). Equal amounts of mouse plasma (70–100 μl) were used for sequential density ultracentrifugation to separate chylomicrons (CM), VLDL (d < 1.006 g/ml), IDL (d 1.006–1.034 g/ml), LDL (d 1.034–1.063 g/ml), and HDL (d 1.063–1.21 g/ml) in a TLA 100 rotor (Beckmann Instruments, Palo Alto, CA). Fractions were used to measure TC and TG, as described above. FPLC used a gel filtration column (GE Superose 6 10/300 GL column) in 0.15 M sodium chloride containing 1 mM ethylenediaminetetraacetic acid and 0.02% sodium azide, pH 7.4. Fractions (0.2 ml) were collected and TC and TG were determined enzymatically as described above. Samples comprising equal amounts of plasma from each of the mice constitute an experimental group.

### Tissue collection:

Mice were deeply anesthetized with xylazine (10 mg/kg) and ketamine (100 mg/kg) and then perfused by heart puncture with 10 ml of phosphate buffered saline (PBS) or until the livers blanched. Tissues were rapidly excised and snap-frozen in liquid nitrogen unless otherwise noted.

### Atherosclerotic lesion analysis:

Each anesthetized mouse was perfused with PBS. The aorta was then exposed and fat was carefully cleaned under a binocular microscope. Pictures of the aortic arch and BCA were taken using a camera fitted to the binocular microscope. The BCA was collected in 10% formalin, kept overnight at 4°C, and then stored in 70% ethanol at 4°C for further processing. The root of the heart was cut and embedded in TissueTek Optimal Cutting Temperature (OCT), frozen, and stored at −80°C.

Serial sections (6 μm) of roots were obtained by cryosectioning. CD68 immunostaining was used to determine macrophage content, as described previously^56–58^. Oil Red O immunostaining was used to determine neutral lipids. Moreover, Picrosirius Red staining was used to assess the collagen content under polarized light. ImageJ 1.53t software (National Institutes of Health) was used for all the quantifications.

### BCA analysis:

BCAs embedded in paraffin were sectioned (5 μm) throughout the entire length of the BCA. Every 5th cross section was stained using the Movat’s pentachrome to visualize atherosclerotic lesions. The maximal lesion site was determined, and adjacent sections were immunostained using a Mac-2 antibody (Cedarlane Lab, catalog CL8942AP; biotinylated secondary antibody Goat-anti-Rat from Southern Biotech, cat. 3050–08) to detect lesion macrophages, as described previously^59^. We used a rat IgG2a isotype control antibody (Cedarlane Lab; catalog CLCR2A00) as a negative control for Mac-2 staining. Morphometric measurements were performed on digitized images of stained serial sections by using IMAGE J, by an investigator blinded to the study groups.

### Circulating and arterial WBCs:

For blood leukocytes, an aliquot of whole blood obtained from retro-orbital plexus of mice was placed into EDTA-coated tubes to prevent coagulation and kept on ice to prevent leukocyte activation. A 30 μl aliquot was used for automated cell counting with remaining cells subjected to red blood cell (RBC) lysis. A portion of white blood cells (WBCs) were then washed and stained with an antibody cocktail containing CD45-AF700 (BD Biosciences, cat# 560510), CD115-APC (Invitrogen, cat# 17–1152-82), Ly6G-eFluor450 (Invitrogen, cat# 48–9668-82), CD3e-FITC (Invitrogen, cat# 11–0031-82) and CD19-PE (Biolegend, cat# 152407) for 30 min in the dark on ice. Antibodies were diluted 1:200. Gated from the CD45+ leukocytes, monocytes were identified as CD115+ and includes the Ly6G low subset. Neutrophils were identified as CD115− and Ly6-G high. T- and B-lymphocytes were identified as CD3+CD19− and CD19+CD3− respectively. All samples were run on a MoFlo XDP sorter (Beckman Coulter) and analyzed using FlowJo (TreeStar Inc, USA). Blood leukocytes are expressed as a percentage of CD45+ cells.

To obtain vascular WBCs, aortic arches were isolated from the mouse and digested in liberase (5 mg/ml; Roche, 273582), DNase I (58 ug/ml; Sigma, DN25) and hyaluronidase (99 ug/ml; Sigma, 3506) for 15min at 37C° using the gentleMacs dissociator (Miltenyi Biotec). Aortic cell suspensions were live/dead stained with Fixable Green Dead Cell Stain Kit (Invitrogen, cat# L34970) and incubated with following antibodies Siglec-F-APC-Cy7 (BD Biosciences, cat#565527), CD45-PerCP-Cy5.5 (Biolegend, cat# 103132), CD45R/B220 (Biolegend, cat#103251), Ly-6G-PE (BD Biosciences, cat# 561104), CD3-APC (Biolegend, cat# 100236), CD11b-BV605 (Biolegend, cat# 101237). All samples were run on a MACSQuant Analyzer 16 (Miltenyi Biotech) and analyzed with FlowJo V10 (Tree-Star). Unstained cells and FMO controls were used as negative controls to set gates. Compensation beads (01–1111-42, ThermoFisher) were used to set compensation.

### Tissue processing of murine aorta and Fluorescence-activated cell sorting (FACS) analysis.

Animals were administered ketamine–xylazine for long anesthetic effect and were perfused thoroughly with PBS. Aortas were dissected and maintained in HypoThermosol^®^ FRS Preservation Solution buffer (Sigma, H4416–100ML) with transcription inhibitor Flavopiridol (Alvocidib, Selleckchem, S1230) on ice till processing. Aortas were chopped in a petri dish and digested for 15 mins at 37 C in 1mg/ml collagenase I (GIBCO, Life Technologies, NY, USA), 1mg/ml, Dispase (GIBCO, Life Technologies, NY, USA), 150 mg/ml DNase-I (Sigma-Aldrich, St Louis, MO, USA), Liberase^™^ TL Research Grade (Sigma, 5401020001) before passing the suspension through a 100 mM cell strainer. Dissociated single cells were stained with live/dead dye in FACS buffer. FACS was performed on a Hasp1 SY32000 (100um) cell sorter. Doublets and debris were gated out and dead cells based on immunofluorescence signal analysis. Live singlets were sorted for single cell sequencing in GTC NYU core.

### Single-cell RNA sequencing analysis.

FACS-sorted single-cell suspensions of LpL-LDLR double knock-out mice and wild-type mice were used to generate barcoded single-cell cDNA libraries for each sample pool PIPseq^™^ V T20 3’ Single Cell RNA Kit (Fluent biosciences) that profiles up to 20,000 cells per reaction. Sequence data from three scRNA-seq libraries constructed from one pool of 4 mouse aorta samples out of 4 libraries and were combined for downstream processing and analysis. Multiple samples were labeled by using four TotalSeq^™^-A Hashtag reagents of BioLegend for each library (TotalSeq^™^-A0301 anti-mouse Hashtag 1 Antibody, 155801, TotalSeq^™^-A0302 anti-mouse Hashtag 2 Antibody, 155803, TotalSeq^™^-A0303 anti-mouse Hashtag 3 Antibody, 155805, TotalSeq^™^-A0304 anti-mouse Hashtag 4 Antibody, 155807). Libraries were loaded onto an Illumina NextSeq-600. To process the sequencing data, we used the pipeline of the platform “PIPseekerTM”. The FASTQ processing step results in general statistics pertaining to the quality of barcode matching, list of all the generated barcodes, intermediate FASTQ file(s) to be used by STAR aligner (Spliced Transcripts Alignment to a Reference) for mapping to mouse genome. Aligned reads were filtered for valid cell barcodes and unique molecular identifiers. An aggregated, between-sample normalized gene expression matrix was generated using cell ranger. The aggregated single-cell gene expression data generated by the PIPseeker was used as the input for the analysis pipeline. PIPseeker defaults to five sensitivity levels (1 → 5). We performed the analysis with the higher sensitivity 5 that results in deeper barcodes labeling as cells. The approach was to perform demultiplexing of the samples with hashtag oligos (HTOs). First, we perform a k-medoid clustering on the normalized HTO values, which initially separates cells into K (# of samples) +1 clusters. We calculate a ‘negative’ distribution for HTO. For each HTO, we use the cluster with the lowest average value as the negative group. For each HTO, we fit a negative binomial distribution to the negative cluster. We use the 0.99 quantile of this distribution as a threshold. Based on these thresholds, each cell is classified as positive or negative for each HTO. The whole coding pipeline for clustering of the cells, identification of gene markers, and differential expression analysis was performed by following Seurat (5.1.0) pipeline in Rstudio (R version 4.4.1 (2024–06-14 ucrt)). Important attached packages for the analysis were “dplyr 1.1.4”, “patchwork 1.2.0” and “SeuratObject 5.0.2 sp_2.1–4”.

Differentially expressed genes were annotated into IPA pathways by applying a cut-off for the p-value (p<0.05). Canonical pathways analysis identified the pathways from the QIAGEN Ingenuity Pathway Analysis library of canonical pathways and right-tailed Fisher’s Exact Test was used to calculate a p-value (Qiagen). Significant results were visualized in the R statistical environment (R v4). Singular and Modular Enrichment Analysis of lists of genes into Biological processes networks was performed via the GeneCodis tool^60^.

### Rabbit lipoprotein production.

TRLs were obtained from control and diabetic male New Zealand White rabbits (weight 3.0 kg; age 12 weeks) as was reported by others^3^. The rabbits were obtained from Charles River Laboratories (Wilmington, MA) and were specific pathogen-free. They were housed and fed ad libitum with Teklad Diet 2031 (Inotiv, West Lafayette, IN) and tap water. Nutritional supplementation included timothy hay and fresh vegetables, enrichment was provided, and rabbits were housed in a 12:12h light: dark cycle at 17.8°C and 20°C in Tecniplast R-Suite cages (West Chester, PA). All experimental procedures were approved by the Institutional Animal Care and Use Committee and conducted at an AAALAC International–accredited facility at the U. Louisville. To create TRLs from hypercholesterolemic diabetic rabbits, rabbits were fed Teklad Diet 2031 for 4 weeks, at week two the rabbit was sedated with butorphanol 0.4 mg/kg and midazolam 0.4 mg/kg, and diabetes was induced using Alloxan (Sigma-Aldrich^®^, St. Louis, MO) at 100 mg/kg, reconstituted to 50 mg/mL in sterile saline, and administered intravenously over 5 minutes. The University of Louisville Comparative Medicine Research Unit Veterinary Team provided constant care in the intensive care unit over 48 hours. Successful diabetes induction was confirmed with blood glucose levels maintaining over 300 mg/dL for a week. At week 4, the rabbit was transitioned over a week to a high-cholesterol modified LabDiet^®^ 5321 (Tucson, AZ) consisting of 7.5% corn oil and 0.5% cholesterol. Blood glucose was measured daily for 2 weeks after diabetes induction, followed by weekly measurements. Additionally, after diabetes induction, 3–8 IU of Humulin^®^ N insulin (Eli Lilly and Co., Indianapolis, IN) was administered every day to maintain blood glucose levels at 300–400 mg/dL. Up to 5 mL of blood was collected in weeks 0, 2, 5, and 7 from an auricular vein. Blood from week 2 was collected immediately before diabetes induction. All blood was centrifuged at 6000 rpm for 5 min at 23°C. Serum was aliquoted into 1.5 mL microcentrifuge tubes and stored at −2°C and then shipped overnight to NYU.

### Lipoprotein uptake by cultured ECs:

Mouse ECs cultured on glass coverslips were allowed to reach confluency, then switched to FBS-free medium overnight. Cells were exposed to DiI-labeled chylomicrons isolated from control and high cholesterol diet-fed (with or without alloxan-induced diabetes) rabbits for 30 minutes in FBS-free media. Following treatment, cells were thoroughly washed with PBS and fixed with 10% formalin. Nuclei were highlighted with DAPI. Uptake of DiI-labeled chylomicrons was assessed by confocal microscopy using a Leica SP8 confocal microscope.

### EC transcytosis of rabbit lipoproteins:

Lipoprotein transcytosis was assessed using confluent primary human coronary artery ECs and TIRF microscopy as previously reported in detail^61^. Isolated rabbit lipoproteins were labeled with DiI and then incubated in cold HPMI media for 10 min at 4°C to allow binding. After washing to remove lipoproteins warm HPMI was added. Cells imaged on the live cell imaging stage at 37°C for 2 min before initial image acquisition. Confluent regions of the monolayer were selected after staining with NucBlue Live ReadyProbes Reagent (Thermo Fisher) and TIRF microscopy of the basal membrane was performed to visualize exocytosis. TIRF microscopy was performed on a Leica DMi8 microscope with 63×/1.47 (O) objectives, 405 nm, 488 nm, 561 nm, and 637 nm laser lines, 450/50, 525/50, 600/50, 610/75, and 700/75 emission filters and run with Quorum acquisition software (Quorum, Canada). Microscope settings were kept constant between conditions. For each coverslip, 10–15 videos of 150 frames (100 ms exposure) were captured. Quantification of lipoprotein transcytosis was performed as described previously^61^.

### Data availability:

The datasets generated and analyzed during the current study are available in the National Center for Biotechnology Information Gene Expression Omnibus (GEO) repository (GSE “pending”). In brief, FASTQ files and matrices of the normalized expression values are listed for each sample of this project along with a description of the analysis.

### Statistical analysis:

Data are presented as means ± SEM. Statistical differences were assessed via unpaired Student’s t-test or a one-way or two-way ANOVA using GraphPad Prism 7 where appropriate. A p-value of <0.05 was considered significant.
